# 40-De­oxy-40(*S*)-iodo­rapamycin

**DOI:** 10.1107/S1600536812027547

**Published:** 2012-07-04

**Authors:** Lijun Xie, Jian Zuo, Guoxin Yang, Congshen Zheng, Yuanrong Cheng

**Affiliations:** a Fujian Institute of Microbiology, Fuzhou, Fujian 350007, People’s Republic of China; b Key Laboratory of Marine Chemistry Theory and Technology, Ministry of Education, College of Chemistry and Chemical Engineering, Ocean University of China, Qingdao, Shandong 266100, People’s Republic of China

## Abstract

The title compound, C_51_H_78_INO_12_, contains a 29-membered ring incorporating amide, lactone and ester groups. It contains a total of 15 stereogenic centres. In the crystal, mol­ecules are linked by O—H⋯O hydrogen bonds, forming *C*(8) chains propagating in [100]. A weak intra­molecular O—H⋯O inter­action also occurs.

## Related literature

For general background to rapamycin and its use as an immunosuppressant drug for rejection prevention in organ transplantation, see: Calne *et al.* (1989 ▶). For the anti­cancer properties of rapamycin derivatives, see: Chan (2004 ▶); Sun *et al.* (2005 ▶); Ayral-Kaloustian *et al.* (2010 ▶). For the structures of related compounds, see: White & Swindells (1981 ▶).
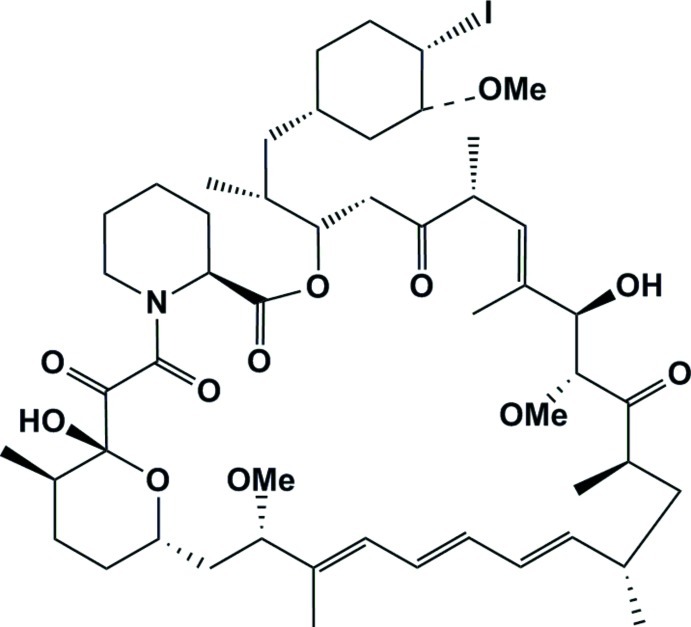


## Experimental

### 

#### Crystal data

C_51_H_78_INO_12_
*M* *_r_* = 1024.04
Orthorhombic, 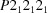

*a* = 12.8905 (2) Å
*b* = 12.9820 (3) Å
*c* = 34.6469 (10) Å
*V* = 5798.0 (2) Å^3^
*Z* = 4
Cu *K*α radiation
μ = 4.77 mm^−1^
*T* = 293 K
0.45 × 0.42 × 0.37 mm

#### Data collection

Bruker SMART CCD diffractometer
Absorption correction: multi-scan (*SADABS*; Bruker, 1996 ▶) *T*_min_ = 0.223, *T*_max_ = 0.271
12528 measured reflections
8657 independent reflections
6937 reflections with *I* > 2σ(*I*)
*R*_int_ = 0.019

#### Refinement

*R*[*F* ^2^ > 2σ(*F* ^2^)] = 0.049
*wR*(*F* ^2^) = 0.136
*S* = 1.03
8657 reflections
596 parameters
H-atom parameters constrained
Δρ_max_ = 0.39 e Å^−3^
Δρ_min_ = −0.51 e Å^−3^
Absolute structure: Flack (1983 ▶), 3167 Friedel pairs
Flack parameter: −0.015 (5)

### 

Data collection: *SMART* (Bruker, 1996) ▶; cell refinement: *SAINT* (Bruker, 1996) ▶; data reduction: *SAINT*; program(s) used to solve structure: *SHELXS97* (Sheldrick, 2008 ▶); program(s) used to refine structure: *SHELXL97* (Sheldrick, 2008 ▶); molecular graphics: *SHELXTL* (Sheldrick, 2008 ▶); software used to prepare material for publication: *SHELXTL*.

## Supplementary Material

Crystal structure: contains datablock(s) I, global. DOI: 10.1107/S1600536812027547/hb6726sup1.cif

Structure factors: contains datablock(s) I. DOI: 10.1107/S1600536812027547/hb6726Isup2.hkl

Additional supplementary materials:  crystallographic information; 3D view; checkCIF report

## Figures and Tables

**Table 1:** Hydrogen-bond geometry (Å, °)

*D*—H⋯*A*	*D*—H	H⋯*A*	*D*⋯*A*	*D*—H⋯*A*
O9—H9⋯O2^i^	0.82	2.12	2.937 (5)	172
O4—H4⋯O6	0.82	2.45	3.150 (7)	144

Symmetry code: (i) 
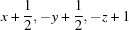
.

**Fig. 1.**
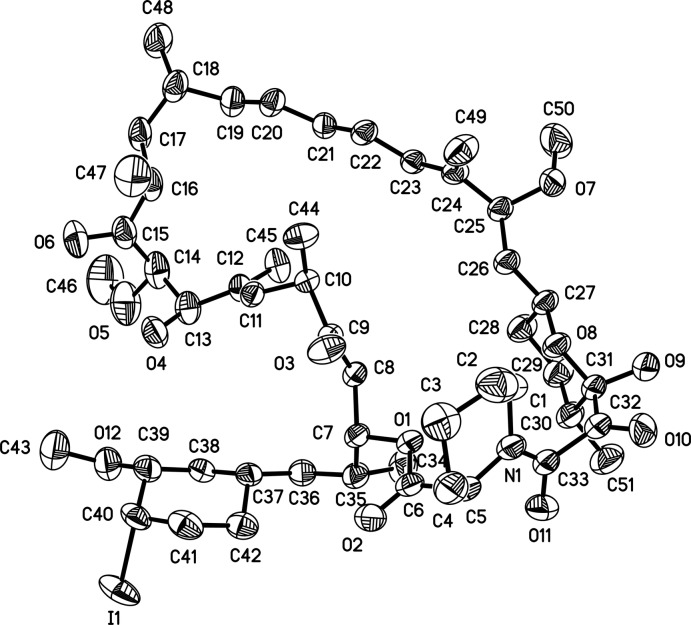
The structure of the title compound, showing 30% probability displacement ellipsoids.

## supplementary crystallographic information

### Comment

Sirolimus is a macrocyclic natural product (also know as rapamycin), has been originally developed as an antifungal agent. However, this application was abandoned when rapamycin become a potent immunosuppressive agent which is used clinically to prevent rejection of transplanted organs (Calne *et al.*, 1989). Recently, there are also many reports about potent anticancer activities of rapamycin derivatives, such as AP-23573, CCI-779 and RAD-001; they are promising agents for treating certain cancers (Chan *et al.*, 2004; Sun *et al.*, 2005). Herein, we present the synthesis and structure of a rapamycin derivative, [40-Deoxy-40(*S*)-iodo]-rapamycin.

The crystal structure of the title compound is given in Fig. 1. The title compound has macrocyclic structure which contains an amide C33—N1, a lactone C6—O1, an oxygen bridge between C27 and C31 and an additional bond between N1 and C5 to form a piperidine unit. In the crystal, the adjacent molecules are stabilized by classical intermolecular O—H···O hydrogen bonding, with the distance of 2.937 (5) Å (Table 1).

### Experimental

Trifluoromethanesulfonic anhydride (21.4 mmol, 3.6 ml) was added gradually to the mixture solution of rapamycin (11.0 mmol, 10 g) and 2,6-Lutidine (65.0 mmol, 7.6 ml) in dry dichloromethane (60 ml). The reaction mixture was stirred at 273 K for 1 h, then the mixture was quenched with a saturated NaHCO3 solution (100 ml) and diluted with dichloromethane. The organic phase was evaporated under reduced pressure to get the crude mixture which dissolved in the mixture of acetone and purified water (50/1), the sodium iodide (55.0 mmol, 9.1 g) was added to the reaction mixture. The reaction mixture was stirred at room temperature for 6 h. The mixture was concentrated under reduced pressure. The crude mixture was purified by flash column chromatography (silica, 1%-10% EtOAc/hexanes) to furnish the product. Colorless blocks of the title compound were obtained in ether solution after 10 day by slow evaporation at room temperature.

### Refinement

All H-atoms were positioned geometrically and refined using a riding model, with C—H = 0.96 Å (CH3), 0.97 Å (CH2), 0.98 Å (CH), and *U*_iso_(H) =1.2*U*_eq_(C).

### Crystal data

**Table:**

C_51_H_78_INO_12_	*F*(000) = 2160
*M**_r_* = 1024.04	*D*_x_ = 1.173 Mg m^−^^3^
Orthorhombic, *P*2_1_2_1_2_1_	Cu *K*α radiation, λ = 1.54178 Å
Hall symbol: P 2ac 2ab	Cell parameters from 4960 reflections
*a* = 12.8905 (2) Å	θ = 3.4–70.6°
*b* = 12.9820 (3) Å	µ = 4.77 mm^−^^1^
*c* = 34.6469 (10) Å	*T* = 293 K
*V* = 5798.0 (2) Å^3^	Block, colorless
*Z* = 4	0.45 × 0.42 × 0.37 mm

### Data collection

**Table:**

Bruker SMART CCD diffractometer	8657 independent reflections
Radiation source: fine-focus sealed tube	6937 reflections with *I* > 2σ(*I*)
Graphite monochromator	*R*_int_ = 0.019
phi and ω scans	θ_max_ = 65.0°, θ_min_ = 3.6°
Absorption correction: multi-scan (*SADABS*; Bruker, 1996)	*h* = −7→15
*T*_min_ = 0.223, *T*_max_ = 0.271	*k* = −15→12
12528 measured reflections	*l* = −40→14

### Refinement

**Table:**

Refinement on *F*^2^	Secondary atom site location: difference Fourier map
Least-squares matrix: full	Hydrogen site location: inferred from neighbouring sites
*R*[*F*^2^ > 2σ(*F*^2^)] = 0.049	H-atom parameters constrained
*wR*(*F*^2^) = 0.136	*w* = 1/[σ^2^(*F*_o_^2^) + (0.0665*P*)^2^ + 2.3571*P*] where *P* = (*F*_o_^2^ + 2*F*_c_^2^)/3
*S* = 1.03	(Δ/σ)_max_ = 0.001
8657 reflections	Δρ_max_ = 0.39 e Å^−^^3^
596 parameters	Δρ_min_ = −0.51 e Å^−^^3^
0 restraints	Absolute structure: Flack (1983), 3167 Friedel pairs
Primary atom site location: structure-invariant direct methods	Flack parameter: −0.015 (5)

### Special details

**Table:**

Geometry. All e.s.d.'s (except the e.s.d. in the dihedral angle between two l.s. planes) are estimated using the full covariance matrix. The cell e.s.d.'s are taken into account individually in the estimation of e.s.d.'s in distances, angles and torsion angles; correlations between e.s.d.'s in cell parameters are only used when they are defined by crystal symmetry. An approximate (isotropic) treatment of cell e.s.d.'s is used for estimating e.s.d.'s involving l.s. planes.
Refinement. Refinement of *F*^2^ against ALL reflections. The weighted *R*-factor *wR* and goodness of fit *S* are based on *F*^2^, conventional *R*-factors *R* are based on *F*, with *F* set to zero for negative *F*^2^. The threshold expression of *F*^2^ > σ(*F*^2^) is used only for calculating *R*-factors(gt) *etc*. and is not relevant to the choice of reflections for refinement. *R*-factors based on *F*^2^ are statistically about twice as large as those based on *F*, and *R*- factors based on ALL data will be even larger.

### Fractional atomic coordinates and isotropic or equivalent isotropic displacement parameters (Å^2^)

**Table:**

	*x*	*y*	*z*	*U*_iso_*/*U*_eq_	
I1	−0.17545 (3)	0.09666 (3)	0.736031 (19)	0.1251 (2)	
N1	0.3544 (3)	0.1997 (4)	0.50916 (12)	0.0826 (13)	
O1	0.2773 (3)	0.2325 (3)	0.58035 (9)	0.0737 (9)	
O2	0.1628 (3)	0.1042 (3)	0.57611 (11)	0.0877 (10)	
O3	0.4124 (3)	0.1311 (3)	0.64312 (12)	0.0934 (11)	
O4	0.2861 (3)	0.2151 (3)	0.78297 (11)	0.1011 (13)	
H4	0.3028	0.1902	0.8038	0.121*	
O5	0.2631 (6)	0.3706 (5)	0.84008 (15)	0.151 (2)	
O6	0.3945 (5)	0.2244 (5)	0.86438 (15)	0.139 (2)	
O7	0.7637 (3)	0.5270 (4)	0.57078 (10)	0.0938 (12)	
O8	0.5009 (3)	0.4141 (3)	0.52099 (8)	0.0763 (9)	
O9	0.4998 (3)	0.5105 (4)	0.46458 (10)	0.0905 (11)	
H9	0.5433	0.4733	0.4544	0.109*	
O10	0.4361 (4)	0.3286 (5)	0.43962 (11)	0.1252 (19)	
O11	0.2345 (3)	0.3083 (4)	0.48592 (13)	0.1094 (15)	
O12	0.0072 (4)	0.1952 (4)	0.78801 (13)	0.1137 (15)	
C1	0.4615 (5)	0.1810 (6)	0.5215 (2)	0.0981 (19)	
H1A	0.4699	0.2035	0.5480	0.118*	
H1B	0.5083	0.2213	0.5055	0.118*	
C2	0.4899 (6)	0.0684 (7)	0.5184 (3)	0.125 (3)	
H2A	0.4932	0.0491	0.4914	0.150*	
H2B	0.5582	0.0578	0.5295	0.150*	
C3	0.4138 (6)	0.0006 (7)	0.5385 (2)	0.116 (2)	
H3A	0.4125	0.0169	0.5658	0.139*	
H3B	0.4340	−0.0709	0.5356	0.139*	
C4	0.3065 (5)	0.0172 (6)	0.52117 (17)	0.0990 (19)	
H4A	0.3066	−0.0057	0.4945	0.119*	
H4B	0.2563	−0.0241	0.5352	0.119*	
C5	0.2745 (5)	0.1295 (5)	0.52284 (15)	0.0855 (17)	
H5	0.2162	0.1367	0.5049	0.103*	
C6	0.2313 (4)	0.1536 (5)	0.56263 (14)	0.0719 (13)	
C7	0.2421 (4)	0.2508 (4)	0.62012 (12)	0.0667 (12)	
H7	0.2265	0.1841	0.6320	0.080*	
C8	0.3355 (4)	0.2973 (4)	0.64068 (13)	0.0660 (11)	
H8A	0.3712	0.3433	0.6231	0.079*	
H8B	0.3114	0.3380	0.6624	0.079*	
C9	0.4104 (4)	0.2181 (4)	0.65501 (13)	0.0620 (11)	
C10	0.4825 (3)	0.2512 (4)	0.68782 (12)	0.0600 (10)	
H10	0.5061	0.3218	0.6830	0.072*	
C11	0.4188 (4)	0.2498 (4)	0.72405 (12)	0.0641 (11)	
H11	0.3911	0.1864	0.7311	0.077*	
C12	0.3979 (4)	0.3274 (4)	0.74680 (13)	0.0695 (12)	
C13	0.3230 (6)	0.3171 (4)	0.78058 (15)	0.0904 (16)	
H13	0.2626	0.3592	0.7738	0.108*	
C14	0.3581 (7)	0.3555 (6)	0.81942 (18)	0.108 (2)	
H14	0.3946	0.4213	0.8165	0.129*	
C15	0.4280 (6)	0.2775 (5)	0.83826 (17)	0.0953 (19)	
C16	0.5400 (6)	0.2697 (5)	0.82599 (15)	0.0909 (18)	
H16	0.5471	0.3049	0.8011	0.109*	
C17	0.6113 (6)	0.3244 (5)	0.85514 (15)	0.096 (2)	
H17A	0.5797	0.3896	0.8622	0.115*	
H17B	0.6151	0.2827	0.8783	0.115*	
C18	0.7203 (6)	0.3450 (5)	0.84130 (15)	0.097 (2)	
H18	0.7531	0.2795	0.8344	0.116*	
C19	0.7174 (5)	0.4132 (5)	0.80634 (14)	0.0845 (16)	
H19	0.6919	0.4797	0.8096	0.101*	
C20	0.7481 (5)	0.3865 (4)	0.77130 (14)	0.0780 (14)	
H20	0.7843	0.3249	0.7688	0.094*	
C21	0.7298 (4)	0.4451 (4)	0.73668 (13)	0.0698 (12)	
H21	0.7016	0.5107	0.7394	0.084*	
C22	0.7503 (4)	0.4126 (4)	0.70110 (13)	0.0702 (12)	
H22	0.7875	0.3516	0.6987	0.084*	
C23	0.7204 (4)	0.4627 (4)	0.66636 (12)	0.0647 (11)	
H23	0.6861	0.5252	0.6693	0.078*	
C24	0.7351 (4)	0.4317 (4)	0.63013 (13)	0.0697 (13)	
C25	0.6875 (4)	0.4936 (4)	0.59765 (12)	0.0714 (12)	
H25	0.6520	0.5538	0.6085	0.086*	
C26	0.6106 (4)	0.4303 (4)	0.57469 (13)	0.0724 (13)	
H26A	0.6479	0.3758	0.5615	0.087*	
H26B	0.5624	0.3981	0.5925	0.087*	
C27	0.5499 (4)	0.4905 (5)	0.54543 (13)	0.0740 (13)	
H27	0.5979	0.5318	0.5299	0.089*	
C28	0.4677 (5)	0.5601 (5)	0.56233 (16)	0.0927 (18)	
H28A	0.5006	0.6154	0.5766	0.111*	
H28B	0.4249	0.5211	0.5801	0.111*	
C29	0.3997 (5)	0.6056 (6)	0.53063 (18)	0.1013 (19)	
H29A	0.3444	0.6457	0.5423	0.122*	
H29B	0.4409	0.6516	0.5148	0.122*	
C30	0.3530 (4)	0.5225 (6)	0.50547 (16)	0.0919 (18)	
H30	0.3086	0.4798	0.5219	0.110*	
C31	0.4411 (4)	0.4533 (5)	0.49015 (14)	0.0791 (15)	
C32	0.4047 (4)	0.3531 (6)	0.47105 (15)	0.0860 (17)	
C33	0.3252 (5)	0.2851 (6)	0.49107 (14)	0.0833 (15)	
C34	0.1586 (6)	0.4208 (6)	0.60107 (19)	0.111 (2)	
H34A	0.2111	0.4590	0.6146	0.166*	
H34B	0.0945	0.4583	0.6018	0.166*	
H34C	0.1796	0.4109	0.5747	0.166*	
C35	0.1438 (4)	0.3156 (5)	0.62051 (15)	0.0783 (14)	
H35	0.0918	0.2782	0.6054	0.094*	
C36	0.1001 (4)	0.3279 (5)	0.66127 (15)	0.0799 (14)	
H36A	0.0313	0.3577	0.6592	0.096*	
H36B	0.1431	0.3771	0.6750	0.096*	
C37	0.0920 (3)	0.2299 (4)	0.68603 (14)	0.0687 (12)	
H37	0.1623	0.2027	0.6895	0.082*	
C38	0.0494 (4)	0.2552 (4)	0.72553 (15)	0.0774 (14)	
H38A	0.0922	0.3079	0.7373	0.093*	
H38B	−0.0202	0.2828	0.7228	0.093*	
C39	0.0461 (4)	0.1630 (5)	0.75147 (17)	0.0886 (17)	
H39	0.1181	0.1413	0.7555	0.106*	
C40	−0.0111 (3)	0.0711 (4)	0.7345 (2)	0.0872 (16)	
H40	0.0048	0.0102	0.7501	0.105*	
C41	0.0243 (4)	0.0518 (4)	0.6939 (2)	0.0920 (18)	
H41A	−0.0215	0.0013	0.6823	0.110*	
H41B	0.0932	0.0220	0.6948	0.110*	
C42	0.0274 (4)	0.1467 (5)	0.66795 (18)	0.0855 (16)	
H42A	0.0565	0.1282	0.6431	0.103*	
H42B	−0.0425	0.1719	0.6638	0.103*	
C43	0.0194 (8)	0.1225 (9)	0.8182 (2)	0.165 (4)	
H43A	−0.0304	0.0681	0.8151	0.248*	
H43B	0.0085	0.1559	0.8426	0.248*	
H43C	0.0882	0.0943	0.8174	0.248*	
C44	0.5762 (4)	0.1815 (5)	0.69079 (19)	0.0928 (17)	
H44A	0.6198	0.2043	0.7116	0.139*	
H44B	0.6143	0.1836	0.6670	0.139*	
H44C	0.5538	0.1121	0.6957	0.139*	
C45	0.4368 (5)	0.4352 (4)	0.74032 (17)	0.0900 (17)	
H45A	0.5072	0.4404	0.7491	0.135*	
H45B	0.3942	0.4828	0.7544	0.135*	
H45C	0.4338	0.4511	0.7133	0.135*	
C46	0.2730 (11)	0.4307 (10)	0.8733 (3)	0.199 (6)	
H46A	0.3361	0.4130	0.8864	0.298*	
H46B	0.2150	0.4182	0.8900	0.298*	
H46C	0.2745	0.5022	0.8662	0.298*	
C47	0.5713 (8)	0.1590 (6)	0.8204 (2)	0.138 (3)	
H47A	0.6335	0.1453	0.8347	0.206*	
H47B	0.5835	0.1463	0.7935	0.206*	
H47C	0.5168	0.1147	0.8294	0.206*	
C48	0.7833 (7)	0.3960 (8)	0.87399 (18)	0.143 (4)	
H48A	0.7533	0.4616	0.8802	0.215*	
H48B	0.8537	0.4057	0.8657	0.215*	
H48C	0.7822	0.3525	0.8964	0.215*	
C49	0.7960 (5)	0.3374 (6)	0.61917 (17)	0.104 (2)	
H49A	0.8452	0.3549	0.5994	0.156*	
H49B	0.7496	0.2855	0.6096	0.156*	
H49C	0.8320	0.3117	0.6414	0.156*	
C50	0.8279 (7)	0.6050 (8)	0.5855 (2)	0.141 (3)	
H50A	0.7867	0.6644	0.5916	0.211*	
H50B	0.8789	0.6232	0.5665	0.211*	
H50C	0.8620	0.5808	0.6084	0.211*	
C51	0.2858 (6)	0.5651 (8)	0.4733 (2)	0.127 (3)	
H51A	0.2256	0.5975	0.4842	0.190*	
H51B	0.2647	0.5099	0.4566	0.190*	
H51C	0.3247	0.6148	0.4587	0.190*	

### Atomic displacement parameters (Å^2^)

**Table:**

	*U*^11^	*U*^22^	*U*^33^	*U*^12^	*U*^13^	*U*^23^
I1	0.05118 (17)	0.0957 (2)	0.2285 (6)	−0.00536 (19)	0.0178 (3)	0.0032 (3)
N1	0.066 (2)	0.122 (4)	0.060 (2)	−0.004 (3)	−0.011 (2)	−0.011 (3)
O1	0.0637 (17)	0.101 (2)	0.0562 (16)	−0.0071 (19)	−0.0120 (15)	−0.0006 (18)
O2	0.072 (2)	0.106 (3)	0.086 (2)	−0.009 (2)	−0.0046 (19)	−0.007 (2)
O3	0.098 (3)	0.081 (2)	0.101 (3)	0.013 (2)	−0.031 (2)	−0.027 (2)
O4	0.109 (3)	0.109 (3)	0.085 (2)	−0.041 (3)	0.013 (2)	0.006 (2)
O5	0.189 (6)	0.161 (5)	0.103 (3)	0.020 (4)	0.064 (4)	−0.007 (3)
O6	0.152 (5)	0.147 (4)	0.118 (3)	−0.047 (4)	0.013 (3)	0.054 (3)
O7	0.088 (2)	0.131 (3)	0.0626 (19)	−0.027 (3)	0.0027 (19)	−0.010 (2)
O8	0.0702 (18)	0.100 (2)	0.0589 (17)	0.001 (2)	−0.0121 (15)	−0.0132 (18)
O9	0.081 (2)	0.120 (3)	0.070 (2)	0.001 (2)	0.0014 (19)	−0.002 (2)
O10	0.126 (4)	0.186 (5)	0.064 (2)	−0.062 (4)	0.021 (2)	−0.032 (3)
O11	0.067 (2)	0.145 (4)	0.116 (3)	−0.011 (3)	−0.021 (2)	0.030 (3)
O12	0.108 (3)	0.134 (4)	0.099 (3)	−0.003 (3)	0.013 (3)	0.025 (3)
C1	0.071 (3)	0.116 (5)	0.107 (4)	0.000 (4)	−0.011 (3)	−0.019 (4)
C2	0.096 (5)	0.142 (7)	0.136 (6)	0.012 (5)	−0.001 (5)	−0.021 (5)
C3	0.112 (5)	0.117 (5)	0.118 (5)	0.012 (5)	0.003 (5)	−0.011 (5)
C4	0.103 (5)	0.118 (5)	0.076 (3)	−0.016 (4)	0.001 (3)	−0.028 (4)
C5	0.073 (3)	0.118 (5)	0.065 (3)	−0.016 (3)	−0.020 (3)	−0.003 (3)
C6	0.057 (3)	0.096 (4)	0.063 (3)	0.003 (3)	−0.015 (2)	0.000 (3)
C7	0.059 (2)	0.087 (3)	0.054 (2)	0.004 (2)	−0.007 (2)	0.002 (2)
C8	0.065 (3)	0.079 (3)	0.054 (2)	0.002 (3)	−0.005 (2)	0.004 (2)
C9	0.061 (2)	0.068 (3)	0.057 (2)	0.005 (2)	−0.001 (2)	−0.001 (2)
C10	0.057 (2)	0.065 (3)	0.058 (2)	0.000 (2)	−0.007 (2)	0.001 (2)
C11	0.073 (3)	0.060 (2)	0.058 (2)	−0.006 (2)	−0.008 (2)	0.007 (2)
C12	0.080 (3)	0.067 (3)	0.061 (3)	−0.006 (2)	0.006 (2)	0.006 (2)
C13	0.112 (4)	0.085 (3)	0.075 (3)	0.002 (4)	0.021 (3)	−0.002 (3)
C14	0.152 (7)	0.093 (4)	0.078 (4)	−0.014 (4)	0.030 (4)	0.001 (4)
C15	0.128 (5)	0.083 (4)	0.074 (3)	−0.040 (4)	0.005 (4)	0.003 (3)
C16	0.136 (5)	0.080 (3)	0.057 (3)	−0.039 (4)	0.001 (3)	−0.001 (3)
C17	0.143 (6)	0.090 (4)	0.055 (3)	−0.053 (4)	−0.005 (3)	0.002 (3)
C18	0.134 (5)	0.095 (4)	0.062 (3)	−0.035 (4)	−0.021 (3)	0.005 (3)
C19	0.113 (4)	0.075 (3)	0.065 (3)	−0.033 (3)	−0.006 (3)	0.000 (3)
C20	0.102 (4)	0.069 (3)	0.063 (3)	−0.023 (3)	−0.020 (3)	0.001 (2)
C21	0.079 (3)	0.070 (3)	0.061 (3)	−0.023 (2)	−0.003 (3)	−0.002 (2)
C22	0.075 (3)	0.072 (3)	0.064 (3)	−0.006 (3)	−0.013 (2)	−0.005 (2)
C23	0.061 (2)	0.074 (3)	0.059 (3)	−0.003 (2)	−0.001 (2)	−0.008 (2)
C24	0.059 (2)	0.092 (4)	0.058 (2)	0.007 (2)	−0.006 (2)	−0.014 (2)
C25	0.068 (3)	0.095 (3)	0.051 (2)	0.002 (3)	−0.005 (2)	−0.014 (2)
C26	0.067 (3)	0.094 (4)	0.056 (2)	0.001 (3)	−0.006 (2)	−0.010 (3)
C27	0.070 (3)	0.100 (4)	0.052 (2)	0.005 (3)	−0.010 (2)	−0.013 (3)
C28	0.087 (4)	0.117 (5)	0.074 (3)	0.019 (4)	−0.009 (3)	−0.015 (3)
C29	0.094 (4)	0.124 (5)	0.085 (4)	0.025 (4)	−0.005 (3)	−0.008 (4)
C30	0.072 (3)	0.137 (5)	0.067 (3)	0.015 (4)	−0.009 (3)	−0.001 (4)
C31	0.067 (3)	0.122 (4)	0.049 (2)	−0.004 (3)	−0.003 (2)	−0.009 (3)
C32	0.072 (3)	0.129 (5)	0.057 (3)	−0.010 (3)	−0.010 (3)	−0.002 (3)
C33	0.067 (3)	0.130 (5)	0.053 (3)	−0.007 (4)	−0.006 (3)	−0.007 (3)
C34	0.108 (5)	0.117 (5)	0.108 (4)	0.031 (4)	0.002 (4)	0.037 (4)
C35	0.066 (3)	0.097 (4)	0.072 (3)	0.012 (3)	−0.006 (2)	0.009 (3)
C36	0.066 (3)	0.090 (4)	0.084 (3)	0.009 (3)	0.000 (3)	0.008 (3)
C37	0.046 (2)	0.079 (3)	0.081 (3)	0.007 (2)	−0.003 (2)	0.010 (3)
C38	0.056 (2)	0.091 (3)	0.085 (3)	−0.009 (2)	−0.001 (2)	0.008 (3)
C39	0.054 (2)	0.109 (4)	0.103 (4)	0.008 (3)	0.006 (3)	0.023 (3)
C40	0.047 (2)	0.081 (3)	0.134 (5)	−0.001 (2)	0.005 (3)	0.026 (4)
C41	0.060 (3)	0.073 (3)	0.144 (5)	0.010 (3)	0.004 (3)	0.004 (4)
C42	0.062 (3)	0.090 (4)	0.105 (4)	0.011 (3)	−0.001 (3)	−0.001 (3)
C43	0.155 (8)	0.218 (11)	0.123 (6)	−0.004 (8)	0.007 (6)	0.065 (7)
C44	0.072 (3)	0.099 (4)	0.107 (4)	0.018 (3)	−0.022 (3)	−0.001 (4)
C45	0.117 (4)	0.064 (3)	0.089 (3)	0.000 (3)	0.032 (4)	−0.001 (3)
C46	0.276 (15)	0.199 (11)	0.120 (6)	0.029 (11)	0.064 (8)	−0.040 (7)
C47	0.193 (9)	0.101 (5)	0.119 (5)	−0.032 (6)	−0.007 (6)	−0.022 (5)
C48	0.168 (7)	0.186 (8)	0.076 (4)	−0.081 (7)	−0.036 (4)	0.014 (5)
C49	0.102 (5)	0.128 (5)	0.082 (4)	0.041 (4)	−0.013 (3)	−0.019 (4)
C50	0.136 (6)	0.173 (8)	0.113 (5)	−0.071 (7)	0.009 (5)	−0.011 (6)
C51	0.101 (5)	0.177 (8)	0.103 (4)	0.029 (5)	−0.028 (4)	0.011 (5)

### Geometric parameters (Å, º)

**Table:**

I1—C40	2.145 (5)	C23—C24	1.332 (6)
N1—C33	1.328 (8)	C23—H23	0.9300
N1—C5	1.455 (7)	C24—C49	1.502 (8)
N1—C1	1.466 (7)	C24—C25	1.513 (7)
O1—C6	1.333 (6)	C25—C26	1.513 (7)
O1—C7	1.470 (6)	C25—H25	0.9800
O2—C6	1.187 (6)	C26—C27	1.500 (7)
O3—C9	1.203 (6)	C26—H26A	0.9700
O4—C13	1.410 (7)	C26—H26B	0.9700
O4—H4	0.8200	C27—C28	1.511 (8)
O5—C46	1.395 (10)	C27—H27	0.9800
O5—C14	1.432 (9)	C28—C29	1.524 (8)
O6—C15	1.217 (7)	C28—H28A	0.9700
O7—C50	1.403 (8)	C28—H28B	0.9700
O7—C25	1.422 (6)	C29—C30	1.511 (9)
O8—C31	1.413 (6)	C29—H29A	0.9700
O8—C27	1.450 (6)	C29—H29B	0.9700
O9—C31	1.382 (7)	C30—C51	1.516 (8)
O9—H9	0.8200	C30—C31	1.542 (8)
O10—C32	1.205 (7)	C30—H30	0.9800
O11—C33	1.221 (7)	C31—C32	1.533 (9)
O12—C43	1.417 (10)	C32—C33	1.520 (8)
O12—C39	1.424 (7)	C34—C35	1.534 (9)
C1—C2	1.512 (11)	C34—H34A	0.9600
C1—H1A	0.9700	C34—H34B	0.9600
C1—H1B	0.9700	C34—H34C	0.9600
C2—C3	1.491 (11)	C35—C36	1.529 (7)
C2—H2A	0.9700	C35—H35	0.9800
C2—H2B	0.9700	C36—C37	1.537 (7)
C3—C4	1.523 (10)	C36—H36A	0.9700
C3—H3A	0.9700	C36—H36B	0.9700
C3—H3B	0.9700	C37—C42	1.501 (8)
C4—C5	1.516 (9)	C37—C38	1.511 (7)
C4—H4A	0.9700	C37—H37	0.9800
C4—H4B	0.9700	C38—C39	1.497 (8)
C5—C6	1.519 (8)	C38—H38A	0.9700
C5—H5	0.9800	C38—H38B	0.9700
C7—C35	1.520 (7)	C39—C40	1.521 (8)
C7—C8	1.524 (7)	C39—H39	0.9800
C7—H7	0.9800	C40—C41	1.498 (9)
C8—C9	1.495 (7)	C40—H40	0.9800
C8—H8A	0.9700	C41—C42	1.526 (8)
C8—H8B	0.9700	C41—H41A	0.9700
C9—C10	1.530 (6)	C41—H41B	0.9700
C10—C11	1.500 (6)	C42—H42A	0.9700
C10—C44	1.513 (7)	C42—H42B	0.9700
C10—H10	0.9800	C43—H43A	0.9600
C11—C12	1.307 (6)	C43—H43B	0.9600
C11—H11	0.9300	C43—H43C	0.9600
C12—C45	1.503 (7)	C44—H44A	0.9600
C12—C13	1.523 (7)	C44—H44B	0.9600
C13—C14	1.505 (9)	C44—H44C	0.9600
C13—H13	0.9800	C45—H45A	0.9600
C14—C15	1.505 (10)	C45—H45B	0.9600
C14—H14	0.9800	C45—H45C	0.9600
C15—C16	1.509 (10)	C46—H46A	0.9600
C16—C47	1.506 (10)	C46—H46B	0.9600
C16—C17	1.539 (8)	C46—H46C	0.9600
C16—H16	0.9800	C47—H47A	0.9600
C17—C18	1.509 (9)	C47—H47B	0.9600
C17—H17A	0.9700	C47—H47C	0.9600
C17—H17B	0.9700	C48—H48A	0.9600
C18—C19	1.501 (8)	C48—H48B	0.9600
C18—C48	1.543 (8)	C48—H48C	0.9600
C18—H18	0.9800	C49—H49A	0.9600
C19—C20	1.323 (7)	C49—H49B	0.9600
C19—H19	0.9300	C49—H49C	0.9600
C20—C21	1.440 (7)	C50—H50A	0.9600
C20—H20	0.9300	C50—H50B	0.9600
C21—C22	1.329 (7)	C50—H50C	0.9600
C21—H21	0.9300	C51—H51A	0.9600
C22—C23	1.422 (6)	C51—H51B	0.9600
C22—H22	0.9300	C51—H51C	0.9600
			
C33—N1—C5	118.5 (5)	C27—C28—C29	110.9 (4)
C33—N1—C1	122.9 (5)	C27—C28—H28A	109.5
C5—N1—C1	117.9 (5)	C29—C28—H28A	109.5
C6—O1—C7	114.8 (4)	C27—C28—H28B	109.5
C13—O4—H4	109.5	C29—C28—H28B	109.5
C46—O5—C14	114.2 (8)	H28A—C28—H28B	108.1
C50—O7—C25	113.0 (4)	C30—C29—C28	111.6 (6)
C31—O8—C27	115.6 (4)	C30—C29—H29A	109.3
C31—O9—H9	109.5	C28—C29—H29A	109.3
C43—O12—C39	115.0 (7)	C30—C29—H29B	109.3
N1—C1—C2	111.6 (6)	C28—C29—H29B	109.3
N1—C1—H1A	109.3	H29A—C29—H29B	108.0
C2—C1—H1A	109.3	C29—C30—C51	113.0 (7)
N1—C1—H1B	109.3	C29—C30—C31	108.8 (5)
C2—C1—H1B	109.3	C51—C30—C31	112.3 (5)
H1A—C1—H1B	108.0	C29—C30—H30	107.5
C3—C2—C1	112.3 (7)	C51—C30—H30	107.5
C3—C2—H2A	109.2	C31—C30—H30	107.5
C1—C2—H2A	109.2	O9—C31—O8	112.3 (4)
C3—C2—H2B	109.2	O9—C31—C32	110.3 (4)
C1—C2—H2B	109.2	O8—C31—C32	100.8 (5)
H2A—C2—H2B	107.9	O9—C31—C30	108.1 (5)
C2—C3—C4	109.3 (6)	O8—C31—C30	110.6 (4)
C2—C3—H3A	109.8	C32—C31—C30	114.7 (5)
C4—C3—H3A	109.8	O10—C32—C33	119.1 (6)
C2—C3—H3B	109.8	O10—C32—C31	120.8 (6)
C4—C3—H3B	109.8	C33—C32—C31	120.2 (5)
H3A—C3—H3B	108.3	O11—C33—N1	123.1 (6)
C5—C4—C3	111.6 (6)	O11—C33—C32	115.9 (6)
C5—C4—H4A	109.3	N1—C33—C32	120.6 (5)
C3—C4—H4A	109.3	C35—C34—H34A	109.5
C5—C4—H4B	109.3	C35—C34—H34B	109.5
C3—C4—H4B	109.3	H34A—C34—H34B	109.5
H4A—C4—H4B	108.0	C35—C34—H34C	109.5
N1—C5—C4	113.4 (5)	H34A—C34—H34C	109.5
N1—C5—C6	115.2 (5)	H34B—C34—H34C	109.5
C4—C5—C6	109.4 (5)	C7—C35—C36	111.9 (4)
N1—C5—H5	106.0	C7—C35—C34	112.7 (5)
C4—C5—H5	106.0	C36—C35—C34	111.0 (5)
C6—C5—H5	106.0	C7—C35—H35	107.0
O2—C6—O1	124.4 (5)	C36—C35—H35	107.0
O2—C6—C5	121.2 (5)	C34—C35—H35	107.0
O1—C6—C5	114.5 (5)	C35—C36—C37	117.0 (5)
O1—C7—C35	110.8 (4)	C35—C36—H36A	108.0
O1—C7—C8	105.0 (4)	C37—C36—H36A	108.0
C35—C7—C8	115.8 (4)	C35—C36—H36B	108.0
O1—C7—H7	108.4	C37—C36—H36B	108.0
C35—C7—H7	108.4	H36A—C36—H36B	107.3
C8—C7—H7	108.4	C42—C37—C38	109.4 (4)
C9—C8—C7	113.2 (4)	C42—C37—C36	113.6 (4)
C9—C8—H8A	108.9	C38—C37—C36	110.5 (4)
C7—C8—H8A	108.9	C42—C37—H37	107.7
C9—C8—H8B	108.9	C38—C37—H37	107.7
C7—C8—H8B	108.9	C36—C37—H37	107.7
H8A—C8—H8B	107.8	C39—C38—C37	112.4 (5)
O3—C9—C8	123.1 (4)	C39—C38—H38A	109.1
O3—C9—C10	120.4 (4)	C37—C38—H38A	109.1
C8—C9—C10	116.5 (4)	C39—C38—H38B	109.1
C11—C10—C44	111.9 (4)	C37—C38—H38B	109.1
C11—C10—C9	106.6 (4)	H38A—C38—H38B	107.9
C44—C10—C9	111.6 (4)	O12—C39—C38	108.0 (5)
C11—C10—H10	108.9	O12—C39—C40	113.8 (5)
C44—C10—H10	108.9	C38—C39—C40	114.1 (5)
C9—C10—H10	108.9	O12—C39—H39	106.8
C12—C11—C10	127.4 (4)	C38—C39—H39	106.8
C12—C11—H11	116.3	C40—C39—H39	106.8
C10—C11—H11	116.3	C41—C40—C39	110.3 (5)
C11—C12—C45	124.0 (4)	C41—C40—I1	110.5 (4)
C11—C12—C13	121.8 (5)	C39—C40—I1	110.4 (4)
C45—C12—C13	114.1 (5)	C41—C40—H40	108.5
O4—C13—C14	111.1 (5)	C39—C40—H40	108.5
O4—C13—C12	110.0 (4)	I1—C40—H40	108.5
C14—C13—C12	117.8 (6)	C40—C41—C42	115.2 (5)
O4—C13—H13	105.6	C40—C41—H41A	108.5
C14—C13—H13	105.6	C42—C41—H41A	108.5
C12—C13—H13	105.6	C40—C41—H41B	108.5
O5—C14—C13	103.6 (6)	C42—C41—H41B	108.5
O5—C14—C15	112.8 (5)	H41A—C41—H41B	107.5
C13—C14—C15	110.1 (6)	C37—C42—C41	110.5 (5)
O5—C14—H14	110.0	C37—C42—H42A	109.6
C13—C14—H14	110.0	C41—C42—H42A	109.6
C15—C14—H14	110.0	C37—C42—H42B	109.6
O6—C15—C14	119.5 (7)	C41—C42—H42B	109.6
O6—C15—C16	120.7 (7)	H42A—C42—H42B	108.1
C14—C15—C16	119.7 (5)	O12—C43—H43A	109.5
C47—C16—C15	110.9 (6)	O12—C43—H43B	109.5
C47—C16—C17	111.4 (6)	H43A—C43—H43B	109.5
C15—C16—C17	110.9 (5)	O12—C43—H43C	109.5
C47—C16—H16	107.8	H43A—C43—H43C	109.5
C15—C16—H16	107.8	H43B—C43—H43C	109.5
C17—C16—H16	107.8	C10—C44—H44A	109.5
C18—C17—C16	115.4 (5)	C10—C44—H44B	109.5
C18—C17—H17A	108.4	H44A—C44—H44B	109.5
C16—C17—H17A	108.4	C10—C44—H44C	109.5
C18—C17—H17B	108.4	H44A—C44—H44C	109.5
C16—C17—H17B	108.4	H44B—C44—H44C	109.5
H17A—C17—H17B	107.5	C12—C45—H45A	109.5
C19—C18—C17	109.7 (6)	C12—C45—H45B	109.5
C19—C18—C48	110.6 (5)	H45A—C45—H45B	109.5
C17—C18—C48	109.5 (5)	C12—C45—H45C	109.5
C19—C18—H18	109.0	H45A—C45—H45C	109.5
C17—C18—H18	109.0	H45B—C45—H45C	109.5
C48—C18—H18	109.0	O5—C46—H46A	109.5
C20—C19—C18	125.3 (6)	O5—C46—H46B	109.5
C20—C19—H19	117.3	H46A—C46—H46B	109.5
C18—C19—H19	117.3	O5—C46—H46C	109.5
C19—C20—C21	125.2 (6)	H46A—C46—H46C	109.5
C19—C20—H20	117.4	H46B—C46—H46C	109.5
C21—C20—H20	117.4	C16—C47—H47A	109.5
C22—C21—C20	124.9 (5)	C16—C47—H47B	109.5
C22—C21—H21	117.6	H47A—C47—H47B	109.5
C20—C21—H21	117.6	C16—C47—H47C	109.5
C21—C22—C23	125.9 (5)	H47A—C47—H47C	109.5
C21—C22—H22	117.1	H47B—C47—H47C	109.5
C23—C22—H22	117.1	C18—C48—H48A	109.5
C24—C23—C22	128.4 (5)	C18—C48—H48B	109.5
C24—C23—H23	115.8	H48A—C48—H48B	109.5
C22—C23—H23	115.8	C18—C48—H48C	109.5
C23—C24—C49	124.0 (5)	H48A—C48—H48C	109.5
C23—C24—C25	118.8 (4)	H48B—C48—H48C	109.5
C49—C24—C25	117.2 (4)	C24—C49—H49A	109.5
O7—C25—C26	105.9 (4)	C24—C49—H49B	109.5
O7—C25—C24	111.6 (4)	H49A—C49—H49B	109.5
C26—C25—C24	111.6 (5)	C24—C49—H49C	109.5
O7—C25—H25	109.2	H49A—C49—H49C	109.5
C26—C25—H25	109.2	H49B—C49—H49C	109.5
C24—C25—H25	109.2	O7—C50—H50A	109.5
C27—C26—C25	114.4 (5)	O7—C50—H50B	109.5
C27—C26—H26A	108.7	H50A—C50—H50B	109.5
C25—C26—H26A	108.7	O7—C50—H50C	109.5
C27—C26—H26B	108.7	H50A—C50—H50C	109.5
C25—C26—H26B	108.7	H50B—C50—H50C	109.5
H26A—C26—H26B	107.6	C30—C51—H51A	109.5
O8—C27—C26	105.4 (4)	C30—C51—H51B	109.5
O8—C27—C28	109.3 (4)	H51A—C51—H51B	109.5
C26—C27—C28	114.6 (4)	C30—C51—H51C	109.5
O8—C27—H27	109.2	H51A—C51—H51C	109.5
C26—C27—H27	109.2	H51B—C51—H51C	109.5
C28—C27—H27	109.2		
			
C33—N1—C1—C2	−145.5 (6)	C50—O7—C25—C24	−71.7 (7)
C5—N1—C1—C2	44.6 (8)	C23—C24—C25—O7	123.2 (5)
N1—C1—C2—C3	−52.4 (9)	C49—C24—C25—O7	−57.3 (6)
C1—C2—C3—C4	58.8 (9)	C23—C24—C25—C26	−118.5 (5)
C2—C3—C4—C5	−55.9 (8)	C49—C24—C25—C26	61.0 (6)
C33—N1—C5—C4	146.4 (5)	O7—C25—C26—C27	−66.1 (5)
C1—N1—C5—C4	−43.2 (7)	C24—C25—C26—C27	172.2 (4)
C33—N1—C5—C6	−86.4 (6)	C31—O8—C27—C26	−178.2 (4)
C1—N1—C5—C6	84.0 (7)	C31—O8—C27—C28	58.2 (6)
C3—C4—C5—N1	47.9 (7)	C25—C26—C27—O8	166.8 (4)
C3—C4—C5—C6	−82.2 (6)	C25—C26—C27—C28	−73.1 (6)
C7—O1—C6—O2	4.3 (7)	O8—C27—C28—C29	−53.6 (7)
C7—O1—C6—C5	−175.0 (4)	C26—C27—C28—C29	−171.5 (5)
N1—C5—C6—O2	176.9 (5)	C27—C28—C29—C30	54.7 (7)
C4—C5—C6—O2	−54.0 (7)	C28—C29—C30—C51	−179.3 (6)
N1—C5—C6—O1	−3.8 (7)	C28—C29—C30—C31	−53.8 (7)
C4—C5—C6—O1	125.4 (5)	C27—O8—C31—O9	61.6 (6)
C6—O1—C7—C35	−84.0 (5)	C27—O8—C31—C32	179.0 (4)
C6—O1—C7—C8	150.4 (4)	C27—O8—C31—C30	−59.3 (6)
O1—C7—C8—C9	−83.5 (5)	C29—C30—C31—O9	−68.4 (6)
C35—C7—C8—C9	154.1 (4)	C51—C30—C31—O9	57.5 (7)
C7—C8—C9—O3	18.6 (7)	C29—C30—C31—O8	55.0 (7)
C7—C8—C9—C10	−158.0 (4)	C51—C30—C31—O8	−179.1 (6)
O3—C9—C10—C11	−99.9 (5)	C29—C30—C31—C32	168.1 (5)
C8—C9—C10—C11	76.9 (5)	C51—C30—C31—C32	−66.0 (8)
O3—C9—C10—C44	22.5 (7)	O9—C31—C32—O10	7.4 (8)
C8—C9—C10—C44	−160.7 (5)	O8—C31—C32—O10	−111.5 (6)
C44—C10—C11—C12	119.1 (6)	C30—C31—C32—O10	129.7 (6)
C9—C10—C11—C12	−118.7 (5)	O9—C31—C32—C33	−170.4 (5)
C10—C11—C12—C45	−0.7 (9)	O8—C31—C32—C33	70.7 (6)
C10—C11—C12—C13	173.7 (5)	C30—C31—C32—C33	−48.1 (7)
C11—C12—C13—O4	1.6 (8)	C5—N1—C33—O11	1.2 (8)
C45—C12—C13—O4	176.5 (5)	C1—N1—C33—O11	−168.6 (6)
C11—C12—C13—C14	130.4 (6)	C5—N1—C33—C32	−171.0 (5)
C45—C12—C13—C14	−54.8 (8)	C1—N1—C33—C32	19.2 (8)
C46—O5—C14—C13	−164.3 (7)	O10—C32—C33—O11	−93.2 (8)
C46—O5—C14—C15	76.6 (9)	C31—C32—C33—O11	84.7 (7)
O4—C13—C14—O5	−72.4 (7)	O10—C32—C33—N1	79.6 (7)
C12—C13—C14—O5	159.4 (5)	C31—C32—C33—N1	−102.6 (7)
O4—C13—C14—C15	48.5 (8)	O1—C7—C35—C36	174.9 (5)
C12—C13—C14—C15	−79.7 (7)	C8—C7—C35—C36	−65.7 (6)
O5—C14—C15—O6	11.0 (9)	O1—C7—C35—C34	−59.1 (6)
C13—C14—C15—O6	−104.2 (7)	C8—C7—C35—C34	60.2 (6)
O5—C14—C15—C16	−165.9 (5)	C7—C35—C36—C37	−46.8 (7)
C13—C14—C15—C16	78.9 (7)	C34—C35—C36—C37	−173.6 (5)
O6—C15—C16—C47	48.5 (8)	C35—C36—C37—C42	−57.6 (6)
C14—C15—C16—C47	−134.7 (6)	C35—C36—C37—C38	178.9 (4)
O6—C15—C16—C17	−75.8 (8)	C42—C37—C38—C39	57.6 (5)
C14—C15—C16—C17	101.0 (6)	C36—C37—C38—C39	−176.6 (4)
C47—C16—C17—C18	70.7 (7)	C43—O12—C39—C38	−167.4 (6)
C15—C16—C17—C18	−165.3 (6)	C43—O12—C39—C40	64.9 (7)
C16—C17—C18—C19	60.6 (7)	C37—C38—C39—O12	178.7 (4)
C16—C17—C18—C48	−177.8 (6)	C37—C38—C39—C40	−53.7 (6)
C17—C18—C19—C20	−115.3 (7)	O12—C39—C40—C41	172.0 (4)
C48—C18—C19—C20	123.8 (7)	C38—C39—C40—C41	47.5 (6)
C18—C19—C20—C21	169.2 (5)	O12—C39—C40—I1	49.6 (6)
C19—C20—C21—C22	−171.9 (5)	C38—C39—C40—I1	−74.9 (5)
C20—C21—C22—C23	170.9 (5)	C39—C40—C41—C42	−48.3 (6)
C21—C22—C23—C24	−177.1 (5)	I1—C40—C41—C42	74.0 (5)
C22—C23—C24—C49	−5.0 (9)	C38—C37—C42—C41	−56.7 (6)
C22—C23—C24—C25	174.4 (5)	C36—C37—C42—C41	179.3 (4)
C50—O7—C25—C26	166.7 (6)	C40—C41—C42—C37	54.5 (6)

### Hydrogen-bond geometry (Å, º)

**Table:**

*D*—H···*A*	*D*—H	H···*A*	*D*···*A*	*D*—H···*A*
O9—H9···O2^i^	0.82	2.12	2.937 (5)	172
O4—H4···O6	0.82	2.45	3.150 (7)	144

Symmetry code: (i) *x*+1/2, −*y*+1/2, −*z*+1.
